# Biological and molecular interplay between two viruses and powdery and downy mildews in two grapevine cultivars

**DOI:** 10.1038/s41438-020-00413-x

**Published:** 2020-11-01

**Authors:** Giovanna Gilardi, Walter Chitarra, Amedeo Moine, Monica Mezzalama, Paolo Boccacci, Massimo Pugliese, Maria Lodovica Gullino, Giorgio Gambino

**Affiliations:** 1 Centre of Competence for the Innovation in the Agro-Environmental sector (AGROINNOVA), University of Torino, Largo Paolo Braccini 2, 10095 Grugliasco, Italy; 2 Institute for Sustainable Plant Protection, National Research Council (IPSP-CNR), Strada delle Cacce 73, 10135 Torino, Italy; 3 Research Centre for Viticulture and Enology, Council for Agricultural Research and Economics (CREA-VE), Via XXVIII Aprile 26, 31015 Conegliano, Italy; 4 Department of Agricultural, Forest and Food Sciences (DISAFA), University of Torino, Largo Paolo Braccini 2, 10095 Grugliasco, Italy

**Keywords:** Biotic, Plant molecular biology

## Abstract

Grapevine may be affected simultaneously by several pathogens whose complex interplay is largely unknown. We studied the effects of infection by two grapevine viruses on powdery mildew and downy mildew development and the molecular modifications induced in grapevines by their multiple interactions. Grapevine fanleaf virus (GFLV) and grapevine rupestris stem pitting-associated virus (GRSPaV) were transmitted by in vitro-grafting to *Vitis vinifera* cv Nebbiolo and Chardonnay virus-free plantlets regenerated by somatic embryogenesis. Grapevines were then artificially inoculated in the greenhouse with either *Plasmopara viticola* or *Erysiphe necator* spores. GFLV-infected plants showed a reduction in severity of the diseases caused by powdery and downy mildews in comparison to virus-free plants. GFLV induced the overexpression of stilbene synthase genes, pathogenesis-related proteins, and influenced the genes involved in carbohydrate metabolism in grapevine. These transcriptional changes suggest improved innate plant immunity, which makes the GFLV-infected grapevines less susceptible to other biotic attacks. This, however, cannot be extrapolated to GRSPaV as it was unable to promote protection against the fungal/oomycete pathogens. In these multiple interactions, the grapevine genotype seemed to have a crucial role: in ‘Nebbiolo’, the virus-induced molecular changes were different from those observed in ‘Chardonnay’, suggesting that different metabolic pathways may be involved in protection against fungal/oomycete pathogens. These results indicate that complex interactions do exist between grapevine and its different pathogens and represent the first study on a topic that still is largely unexplored.

## Introduction

Grapevine (*Vitis vinifera* L.) is one of the most economically important and widespread fruit crops in the world. In all viticultural regions, it is affected (often simultaneously) by several pathogens, such as fungi, oomycota, phytoplasma, and viruses, which induce serious damage to the plants with consequent high economic losses^1^. Among these biotic agents, viruses represent a widespread class of pathogens in all grapevine species. Thanks to deep sequencing technologies and the subsequent discovery of new viruses, more than 80 virus species have been identified in grapevine^2^. Among these, about half are recognized as agents of ‘major diseases’ in terms of economic relevance^3^, while others may not show clear, typical symptoms possibly also for mixed infections with other viruses in the same plant. Two well known and widespread viruses are grapevine rupestris stem pitting-associated virus (GRSPaV) and grapevine fanleaf virus (GFLV). GRSPaV is a member of the genus *Foveavirus*, family *Betaflexiviridae*, generally associated with “Rupestris Stem Pitting”, a disorder of the “Rugose Wood complex”^3^, and it usually infects *V. vinifera* cultivars in a latent state. GFLV is a member of the genus *Nepovirus* in the family *Secoviridae*^4^ responsible for the complex of ‘Grapevine infectious degeneration’ and is one of the most harmful and economically impacting grapevine–virus diseases worldwide^3^. Several studies have analysed the ecophysiological, molecular, and biochemical modifications induced by viruses in grapevine^5–10^, which can then be regarded as a model to study plant–virus interactions in fruit plants and other woody species^11^. In recent years, it has become increasingly evident that plant–virus interactions are not limited to a simple reaction to pathogen infection and that they can shift from antagonist to mutualistic or beneficial responses to environmental changes^12,13^. Viruses can increase the tolerance of plants to drought and cold stress^14^, high soil temperatures^15^, and bacterial pathogens^16,17^. *V. vinifera* cv ‘Bosco’ infected by GRSPaV showed greater tolerance to water stress than virus-free plants^18^. The detrimental effects of grapevine leafroll associated virus-3 (GLRaV-3) have been extensively demonstrated in grapevine^5,8^ although Repetto et al.^19^ reported that GLRaV-3-infected grapevines displayed an increased resistance to downy mildew (DM) caused by *Plasmopara viticola*. All together these studies show that plant–virus interactions are more complex than expected and a deeper understanding of these interactions would be warranted, considering the plant as a holobiont in which micro- and macrobiomes interact with the environment as a unique organism^20^.

If viruses are the most numerous and widespread class of pathogens in all *Vitis* spp., doubtless, fungal/oomycete-associated diseases represent, in terms of economic and environmental impact, the main adversity in the vineyard, which can lead to a total loss of production if not efficiently controlled. In European grape-growing regions, the most important pathogens significantly impacting crop quality and quantity are DM and powdery mildew (PM). DM is caused by the obligate biotrophic oomycete *P. viticola*, introduced into Europe from North America in the late 19th century^21^. DM affects the leaves and berries under warm and humid environmental conditions, reducing grape yield and negatively impacting the quality of berries and the aging ability of wine^1,22^. PM is caused by the obligate biotrophic fungus *Erysiphe necator*, a polycyclic fungus infecting all green tissues with grey-white mycelia on the surface, leading to significant losses in yield and quality, as well as fruit and wine characteristics^1,23^. Management of these diseases in the vineyard implicates significant economic and environmental costs, to such an extent that grapevines are one of the most frequently sprayed crops^24–26^. The European Commission has already expressed its recommendation to reduce the use of several chemical products (Directive 2009/128/EC), including copper salts (CE no. 354/2014). Consequently, in recent years, several studies have been carried out to investigate the effectiveness of more environmentally friendly products, such as several microorganisms, resistance inducers, biostimulants, and biofungicides^27–30^. However, so far, no studies have been reported on the induction of resistance to PM by viruses while only a few studies have been carried out on DM^19^.

The aim of this work was to study the effects of GRSPaV and GFLV on DM and PM incidence and severity and the systemic molecular modifications induced in grapevines by these multiple interactions under controlled greenhouse conditions.

## Results

### Virus identification and plant material

In the present study the interactions among viruses, DM and PM were evaluated on ‘Chardonnay’ and ‘Nebbiolo’ grapevine cultivar. Immature anthers of both genotypes were collected in May 2016 and cultured in an inductive medium for embryogenesis to obtain virus-free plants, as confirmed by RT-PCR. A single line per cultivar regenerated from somatic embryos was multiplied in vitro, thus supplying the virus-free controls (NE_CTR and CH_CTR) for subsequent experiments (Supplementary Fig. S1).

A ‘Nebbiolo’ plant originally infected only by GFLV and GRSPaV was previously subjected to sanitation techniques that eliminated GFLV in some explants and GRSPaV in other explants, thus obtaining lines still infected by only one of these two viruses. To identify the GRSPaV variant present in infected plant, the viral RNA-dependent RNA polymerase (RdRP) was amplified by RT-PCR^31^ and sequenced. The GRSPaV isolate (GRSPaV_NE, NCBI accession number MN889892) belongs to the phylogenetic group designed as GRSPaV-SG1 (Supplementary Fig. S2a), following the classification in the eight major groups suggested by Meng and Rowhani^32^. The GFLV variant infecting ‘Nebbiolo’ was identified by RT-PCR^33^ by amplification of the RdRP sequence in RNA1 and by sequencing. The GFLV isolate (GFLV_NE, NCBI accession number MN889891) grouped together in the cluster IB with isolates from Canada and South Africa (Supplementary Fig. S2b).

‘Nebbiolo’ plants infected by GRSPaV or GFLV were then used as “green rootstocks” for the transmission of these viruses through in vitro grafting in virus-free lines of ‘Chardonnay’ and ‘Nebbiolo’, used as scions. One month after grafting, viral diagnoses confirmed the transmission of the viruses and the infected scions of ‘Chardonnay’ and ‘Nebbiolo’ were removed from the graft and multiplied in vitro becoming the virus-infected treatment (NE_GRSPaV, NE_GFLV, CH_GRSPaV, and CH_GFLV) for subsequent experiments (Supplementary Fig. S1).

### Development of powdery and downy mildews

Virus-free and infected plants (eight plants per treatment) were grown in the glasshouse and, after 2 months, artificially inoculated with *P. viticola* or *E. necator* for the first trial of the experiment (June 2018). The percentage of affected leaves and the percentage of affected leaf area, i.e. disease incidence (DI) and severity (DS), respectively, were evaluated. At the final assessment, higher levels of downy mildew DS were observed in ‘Chardonnay’ compared to ‘Nebbiolo’, whereas in plants infected by *E. necator*, ‘Chardonnay’ appeared to be more tolerant than ‘Nebbiolo’ to PM. Plant genotype, virus, and their interaction significantly (*p* ≤ 0.05) influenced the DI and the DS caused by both pathogens. GFLV-infected plants showed lower downy mildew DS compared to virus-free plants, while GRSPaV-infected plants showed a DS not significantly different from CTR, with intermediate values between virus-free and GFLV-infected plants (Table 1). A similar trend was observed on plants artificially inoculated with *E. necator*. The genotype and virus interactions (G × V) were significant, resulting in significant lower values of DS in NE_GFLV compared to CTR for both fungal/oomycete pathogens (Table 1).

**Table 1:** Mean disease severity (percentages of affected leaf area) and mean disease incidence (percentage of affected leaves) of ‘Chardonnay’ (CH) and ‘Nebbiolo’ (NE) artificially inoculated with *Plasmopara viticola* and *Erysiphe necator* at the end of trial 1. NE_CTR, CH_CTR: virus-free plants; NE_GRSPaV, CH_GRSPaV: GRSPaV-infected plants; NE_GFLV, CH_GFLV: GFLV-infected plants. Statistical significance values for the factors ‘genotype’, ‘virus’ and their interaction (genotype X virus) for disease severity and disease incidence are reported

		*Plasmopara viticola*		*Erysiphe necator*	
Source of variance		Disease severity %	Disease incidence %	Disease severity %	Disease incidence %
**Genotype**	CH	9.17 ± 3.74 b^a^	59.94 ± 15.62 b	3.72 ± 2.67 a	44.86 ± 18.23 a
	NE	4.83 ± 4.85 a	38.31 ± 17.69 a	6.36 ± 4.05 b	62.39 ± 15.67 b
	*p* value	<0.001	<0.001	0.042	0.019
**Virus**	CTR	10.96 ± 1.69 c	50.34 ± 7.92 b	7.28 ± 1.3 b	58.31 ± 13.96 a
	GRSPaV	6.06 ± 0.99 b	52.62 ± 4.70 b	4.87 ± 4.86 ab	58.47 ± 22.00 a
	GFLV	3.45 ± 1.09 a	35.56 ± 9.12 a	2.70 ± 0.7 a	42.04 ± 16.53 a
	*p* value	<0.001	0.015	0.030	0.157
Genotype*Virus	CH_CTR	13.11 ± 0.91 c	65.04 ± 10.83 b	5.56 ± 1.39 ab	50.10 ± 6.76 ab
	CH_GRSPaV	7.38 ± 1.53 a-c	60.96 ± 6.12 b	3.60 ± 1.06 ab	48.26 ± 8.28 ab
	CH_GFLV	6.27 ± 0.42 a-c	56.84 ± 6.96 b	1.50 ± 0.84 a	32.22 ± 13.48 a
	NE_CTR	7.87 ± 2.59 bc	38.57 ± 8.80 ab	9.00 ± 1.88 b	66.53 ± 4.81 ab
	NE_GRSPaV	5.00 ± 1.22 ab	45.94 ± 5.65 ab	6.42 ± 2.46 ab	71.23 ± 10.52 b
	NE_GFLV	0.63 ± 0.32 a	14.29 ± 6.18 a	3.62 ± 0.81 a	49.43 ± 1.84 ab
	*p* value	0.047	0.001	0.041	0.052

^a^Lowercase letters denote significant differences attested by Tukey’s honestly significant difference (HSD) test (*p* ≤ 0.05). Standard errors are indicated

The trends of susceptibility to PM and DM were confirmed in the second trial conducted in summer 2019, where eight plants were inoculated with *P. viticola* or *E. necator* for each thesis (Table 2). ‘Chardonnay’ was consistently shown to be more susceptible to DM and less susceptible to PM than ‘Nebbiolo’. The GFLV-infected plants showed significantly lower DS for both pathogens and a lower DI for PM compared to CTR when the virus was considered as a source of variance. Interestingly, GRSPaV-infected plants showed, for DM, higher DS than GFLV-infected plants (Table 2). In plants inoculated with *E. necator*, the GFLV-infected plants (of both cultivars) were more tolerant to PM than virus-free controls resulting in lower DS. A similar trend was observed for DI, where CH_GFLV plants showed the significantly lowest values (Table 2).

**Table 2:** Mean disease severity (percentages of affected leaf area) and mean disease incidence (percentage of affected leaves) of ‘Chardonnay’ (CH) and ‘Nebbiolo’ (NE) artificially inoculated with *Plasmopara viticola* and *Erysiphe necator* at the end of trial 2. NE_CTR, CH_CTR: virus-free plants; NE_GRSPaV, CH_GRSPaV: GRSPaV-infected plants; NE_GFLV, CH_GFLV: GFLV-infected plants. Statistical significance values for the factors ‘genotype’, ‘virus’ and their interaction (genotype X virus) for disease severity and disease incidence are reported

		*Plasmopara viticola*		*Erysiphe necator*	
Source of variance		Disease severity %	Disease incidence %	Disease severity %	Disease incidence %
**Genotype**	CH	21.12 ± 2.31 b^a^	65.89 ± 12.42 a	4.39 ± 4.13 a	43.71 ± 24.77 a
	NE	11.90 ± 1.73 a	57.23 ± 20.17 a	6.78 ± 4.11 a	64.12 ± 18.25 b
***p*** **value**		*0.004*	*0.998*	*0.061*	*0.021*
**Virus**	CTR	18.26 ± 2.33 b	55.41 ± 19.35 a	8.35 ± 1.02 c	63.09 ± 5.48 b
	GRSPaV	22.09 ± 3.39b	68.51 ± 18.33 a	5.33 ± 0.99 b	53.65 ± 5.31 b
	GFLV	9.78 ± 1.13 a	60.43 ± 11.47 a	1.64 ± 0.46 a	31.58 ± 6.56 a
***p*** **value**		*0.006*	*0.078*	*<0.001*	*0.001*
Genotype*Virus	CH_CTR	23.38 ± 2.35 bc	67.60 ± 5.43 ab	6.98 ± 1.56 bc	50.99 ± 8.30 b
	CH_GRSPaV	25.58 ± 4.63 c	66.70 ± 4.83 ab	4.95 ± 1.45 a-c	57.92 ± 8.48 b
	CH_GFLV	11.71 ± 1.99 ab	59.60 ± 3.66 ab	0.70 ± 0.20 a	15.98 ± 3.39 a
	NE_CTR	11.09 ± 1.44 ab	50.14 ± 5.52 a	9.72 ± 1.18 c	73.67 ± 5.15 b
	NE_GRSPaV	17.20 ± 4.58 bc	78.85 ± 6.94 b	5.71 ± 1.46 a-c	49.38 ± 6.66 b
	NE_GFLV	8.18 ± 0.97 a	59.60 ± 5.97 ab	2.69 ± 0.73 ab	47.18 ± 9.69 b
***p*** **value**		*0.001*	*0.033*	*<0.001*	*<0.001*

^a^Lowercase letters denote significant differences attested by Tukey’s honestly significant difference (HSD) test (*p* ≤ 0.05). Standard errors are indicated

### Virus titre

Virus titre was monitored in the second trial in 2019 by quantitative real-time RT-PCR (RT-qPCR) in leaf tissue immediately before fungal/oomycete inoculation (T0) and at the end of experiments (Tf), and was calculated as the relative expression ratio to the virus titre in ‘Nebbiolo’ at T0. GRSPaV concentration in ‘Chardonnay’ and ‘Nebbiolo’ was similar at T0 and decreased significantly at Tf (Supplementary Fig. S3). Similarly, the concentration of GFLV decreased considerably at Tf, but its level at T0 and Tf in ‘Nebbiolo’ were 5–6 times lower than in ‘Chardonnay’; the statistical analysis revealed a significant effect of genotype, time, and their interaction (Supplementary Fig. S3).

### Molecular interactions between viruses and powdery and downy mildews

The expression of 15 genes representative of the most important molecular pathways involved in the response of biotic agents in grapevine (Supplementary Table S1) was analysed by RT-qPCR, in leaves collected in the second trial in 2019. To evaluate the viral and fungal/oomycete-mediated systemic responses in virus-infected and fungal/oomycete-inoculated plants, asymptomatic leaves were collected.

**Fig. 1:**
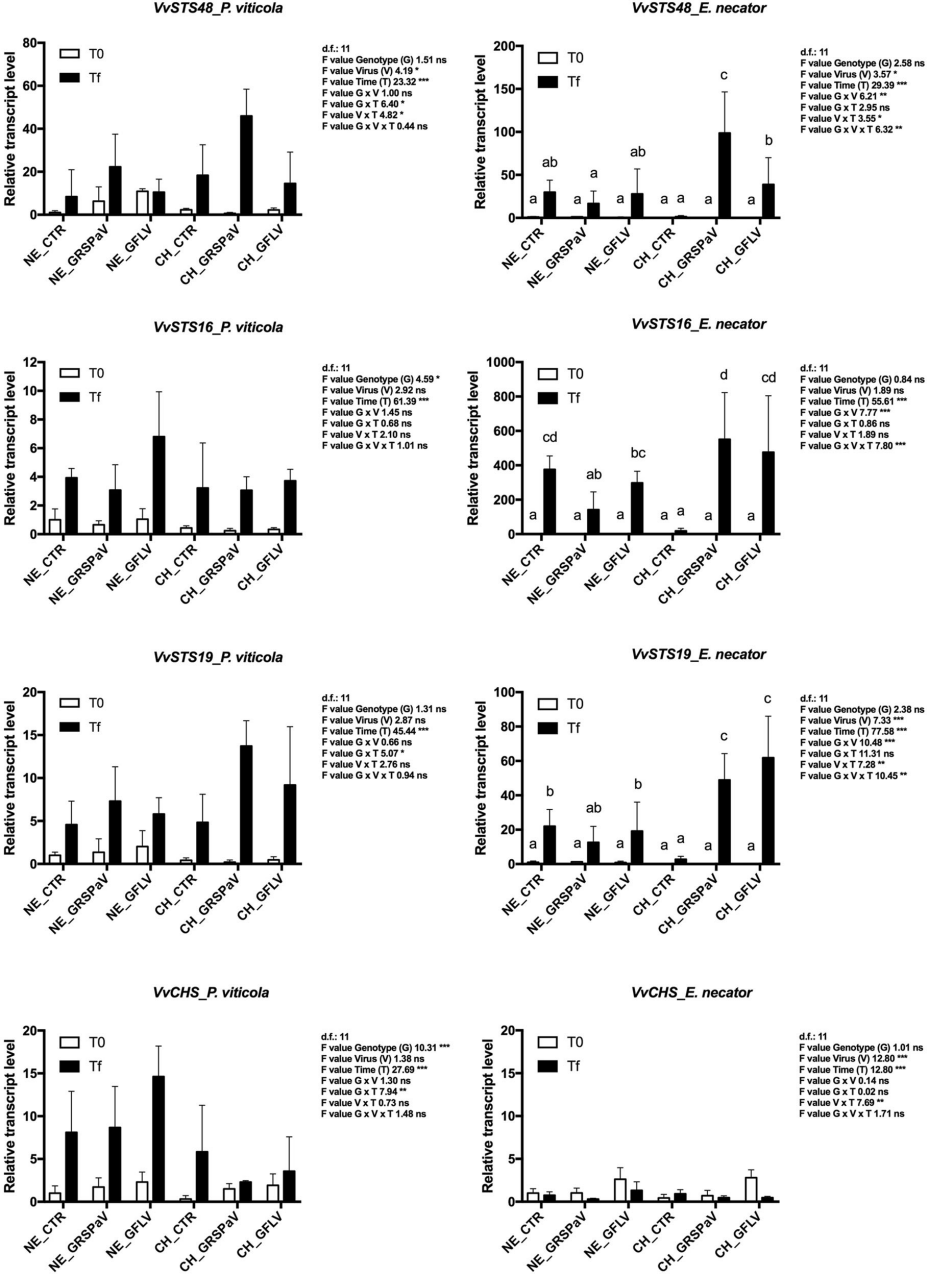
Relative expression levels of *VvSTS48* (VIT_16s0100g01200), *VvSTS16* (VIT_16s0100g00920), *VvSTS19* (VIT_16s0100g00750), and *VvCHS* (VIT_05s0136g00260) measured by quantitative reverse transcription-polymerase chain reaction (RT-qPCR). Samples were collected before inoculation with *P. viticola* or *E. necator* (T0) and at the end of experiments (Tf). RT-qPCR signals were normalized to *VvAct* and *VvUBI* transcripts. NE_CTR, CH_CTR: virus-free plants; NE_GRSPaV, CH_GRSPaV: GRSPaV-infected plants; NE_GFLV, CH_GFLV: GFLV-infected plants. Data are presented as the mean ± standard deviation (SD) (n = 3). Lowercase letters denote significant differences attested by Tukey’s honestly significant difference (HSD) test (*p* ≤ 0.05)

Stilbene synthases are a class of genes of the phenylpropanoid metabolism and are strongly involved in plant responses to environmental stresses, and in grapevine *VvSTS48*, *VvSTS16*, and *VvSTS19* (Fig. 1) are involved in the response to fungal pathogens^34^. All three genes were overexpressed at Tf and expression levels systemically induced by *E. necator* (Tf_EN) were much higher than those observed after *P. viticola* (Tf_PV) inoculation (Fig. 1). A genotype effect was not observed in the expression of these three genes, while a significant interaction G x V was reported after *E. necator* inoculation with high expression in virus-infected plants of ‘Chardonnay’ (CH_GRSPaV_Tf and CH_GFLV_Tf) (Fig. 1, Supplementary Table S2). Noteworthy, the inoculation of ‘Chardonnay’ with *E. necator* did not seem to be sufficient to activate transcription of these stilbene synthases in the virus-free samples (CH_CTR) (Fig. 1).

The expression of chalcone synthase (*VvCHS*) at Tf was influenced by fungal/oomycete inoculation. After inoculation with *E. necator*, transcription was reduced, whereas in the presence of *P. viticola*, we observed a significant increase in gene expression (Fig. 1, Supplementary Table S2). A significant interaction between genotype and time (G x T) after *P. viticola* inoculation showed an increase in *VvCHS* expression in ‘Nebbiolo’ at Tf (Supplementary Table S3). Interestingly, the expression data of *VvSTSs* and *VvCHS* confirmed the opposite transcriptional regulation trend; high expression of *VvSTSs* at Tf_EN corresponded to the downregulation of *VvCHS*, as well as lower expression of *VvSTS19* in ‘Nebbiolo’ Tf_PV compared to ‘Chardonnay’, which corresponds to an increase in *VvCHS* expression under the same conditions (Fig. 1).

**Fig. 2:**
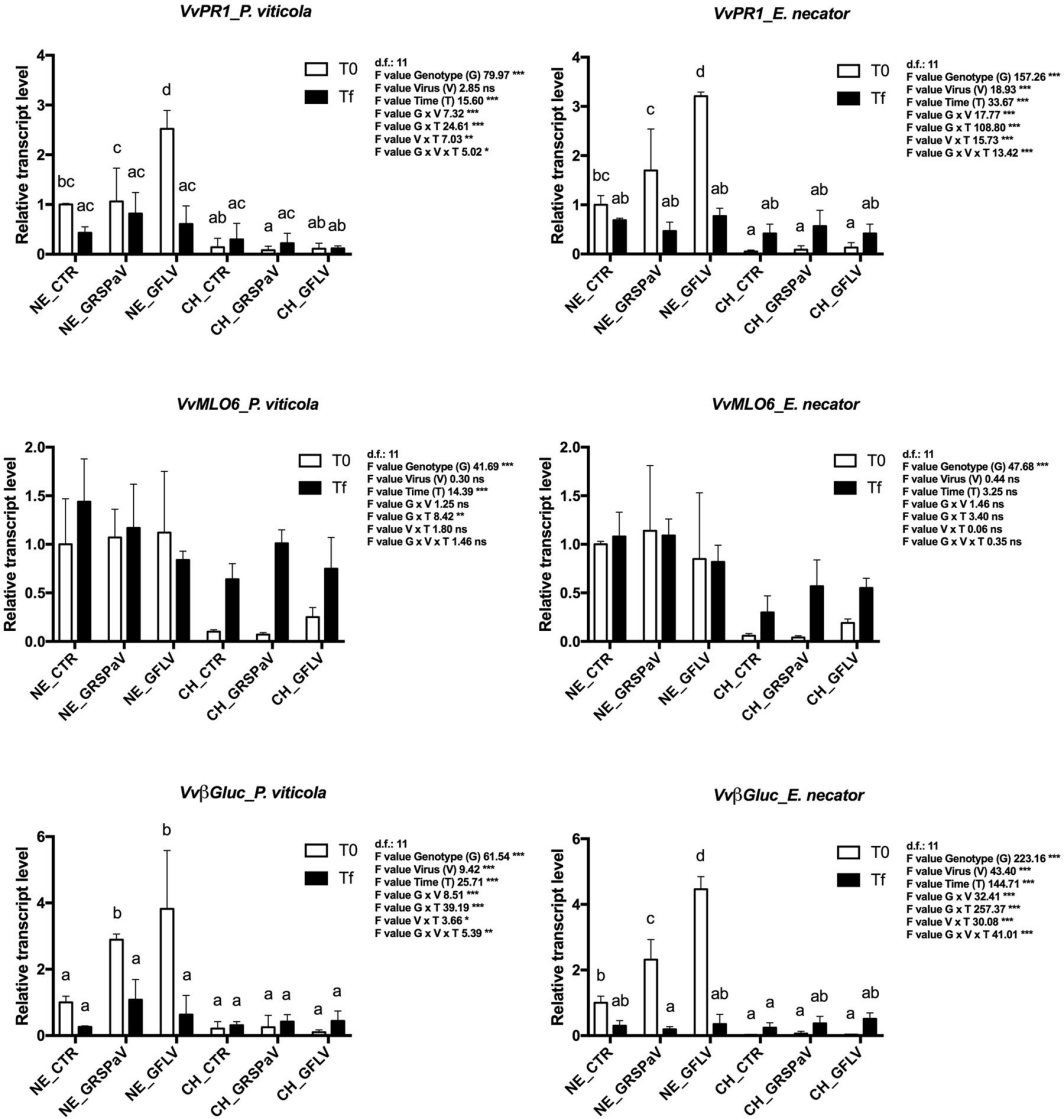
Relative expression levels of *VvPR1* (VIT_03s0088g00700), *VvB gluc* (VIT_08s0007g06060), and *VvMLO6* (VIT_08s0040g02170) measured by quantitative reverse transcription-polymerase chain reaction (RT-qPCR). Samples were collected before inoculation with *P. viticola* or *E. necator* (T0) and at the end of experiments (Tf). RT-qPCR signals were normalized to *VvAct* and *VvUBI* transcripts. NE_CTR, CH_CTR: virus-free plants; NE_GRSPaV, CH_GRSPaV: GRSPaV-infected plants; NE_GFLV, CH_GFLV: GFLV-infected plants. Data are presented as the mean ± standard deviation (SD) (*n* = 3). Lowercase letters denote significant differences attested by Tukey’s honestly significant difference (HSD) test (*p* ≤ 0.05)

The expression of Pathogenesis related protein 1 (*VvPR1*) and Beta-1,3-glucanase (*VvBgluc*), defense-related genes involved in the response to several biotic factors, was significantly affected by all three factors, namely genotype, virus, time, and their respective interactions (Fig. 2, Supplementary Table S2). They were generally upregulated in ‘Nebbiolo’ at T0 and showed an interesting G x V interaction with a progressive increase in the expression of NE_GRSPaV_T0 and NE_GFLV_T0 (Fig. 2, Supplementary Table S2). At Tf, expression was stable in ‘Chardonnay’, while in ‘Nebbiolo’, it decreased with respect to the levels observed at T0 (Fig. 2). The expression of mildew resistance locus O 6 (*VvMLO6*), one of the genes linked to *E. necator* susceptibility, was strongly genotype dependent; the higher expression in ‘Nebbiolo’ at T0 confirmed its higher susceptibility to PM compared to ‘Chardonnay’ (Table 1). At Tf, expression was stable in ‘Nebbiolo’, while it increased in ‘Chardonnay’ regardless of virus or fungal/oomycete infection (Fig. 2).

**Fig. 3:**
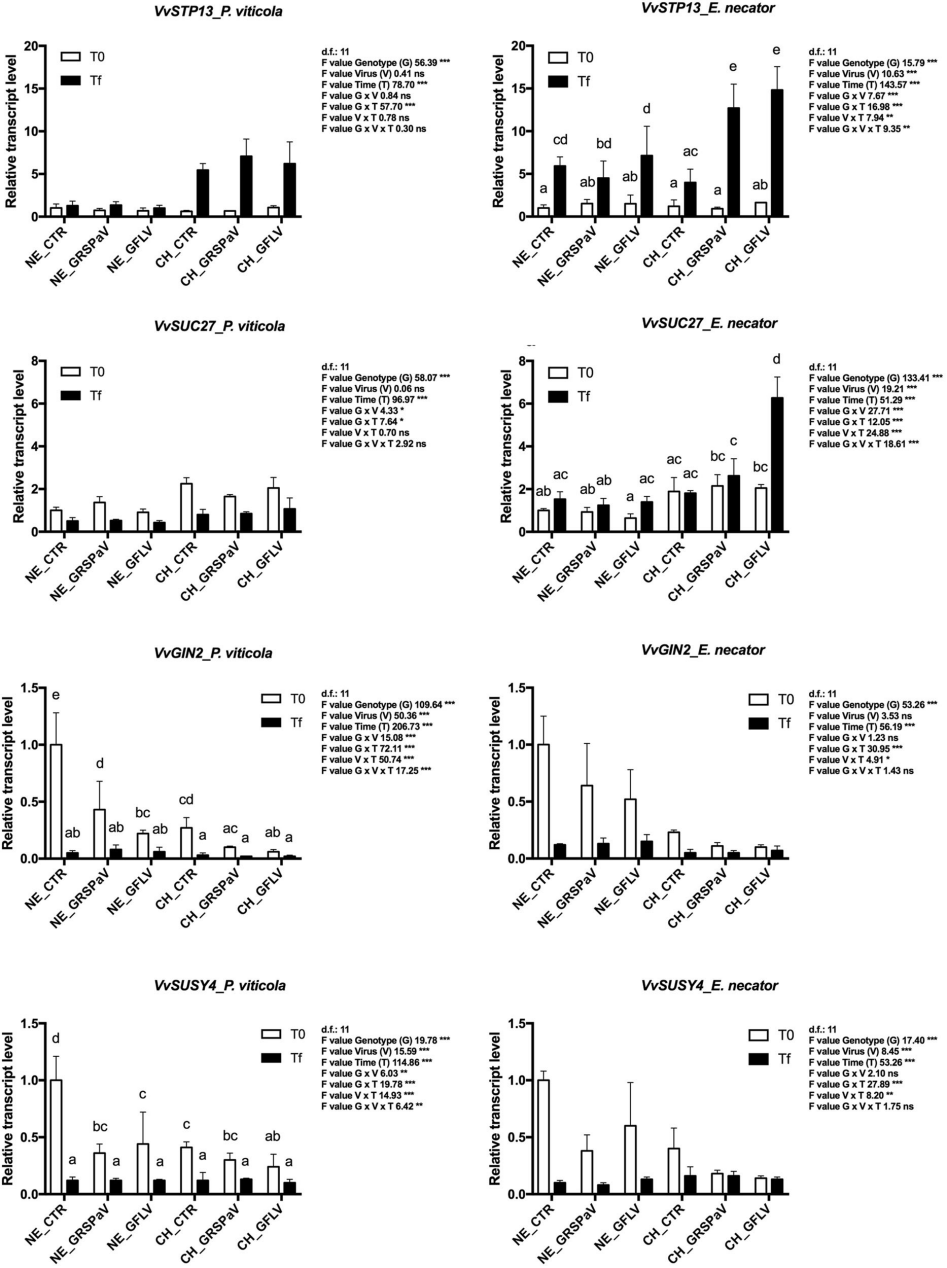
Relative expression levels of *VvSTP13* (VIT_05s0020g03140), *VvSUC27* (VIT_18s0076g00250), *VvGIN2* (VIT_02s0154g00090), and *VvSUSY4* (VIT_11s0016g00470) measured by quantitative reverse transcription-polymerase chain reaction (RT-qPCR). Samples were collected before inoculation with *P. viticola* or *E. necator* (T0) and at the end of experiments (Tf). RT-qPCR signals were normalized to *VvAct* and *VvUBI* transcripts. NE_CTR, CH_CTR: virus-free plants; NE_GRSPaV, CH_GRSPaV: GRSPaV-infected plants; NE_GFLV, CH_GFLV: GFLV-infected plants. Data are presented as the mean ± standard deviation (SD) (*n* = 3). Lowercase letters denote significant differences attested by Tukey’s honestly significant difference (HSD) test (*p* ≤ 0.05)

Another pathway strongly involved in the response to pathogens was found to be carbohydrate metabolism. Sugar transporter 13 (*VvSTP13*) expression was modulated by the multiple interactions among genotype, time (after *P. viticola* inoculation), and viruses (after *E. necator*) (G × V × T). At Tf, expression was higher in ‘Chardonnay’, particularly in the presence of GRSPaV and GFLV (Fig. 3, Supplementary Table S2). Expression of the sucrose-proton symporter 27 (*VvSUC27*) was regulated differentially following fungal/oomycete inoculation. After inoculation with *P. viticola*, the gene was significantly overexpressed in ‘Chardonnay’ and downregulated at Tf while, after *E. necator* inoculation, we observed multiple interactions among all components (G × V × T) with a significant increase in CH_GFLV_TF_En (Fig. 3, Supplementary Table S2). Vacuolar invertase 2 (*VvGIN2*) and sucrose synthase (*VvSUSY4*) showed higher expression levels in ‘Nebbiolo’ at T0, particularly in the absence of viruses (NE_CTR). Infection by viruses and fungus/oomycete tended to downregulate these genes in both cultivars (Fig. 3, Supplementary Table S2).

The transcription factor WRKY18 (*VvWRKY18*), which is involved in activation of stress response pathways, was upregulated at Tf with a significant G × T interaction (Supplementary Fig. S4, Table S2). Whereas, the NAC domain containing protein 17 (*VvNAC17*), a transcription factor involved in the response to several stressors that improve the efficiency of the enzymatic antioxidant defence system^35^, was upregulated at Tf in virus-infected plants (Supplementary Fig. S4). The 9-cis-epoxycarotenoid dioxygenase 1(*VvNCED1*), a key gene in abscisic acid synthesis, was upregulated only at Tf in ‘Nebbiolo’, particularly after *P. viticola* inoculation, while in ‘Chardonnay’ was stable under all conditions (Supplementary Fig. S4). Finally, 12-oxophytodienoate reductase 3 (*VvOPR3*), a gene linked to jasmonate biosynthesis, had an expression independent on both the genotype and virus infection; it was significantly overexpressed at Tf (Supplementary Fig. S4, Table S2).

**Fig. 4:**
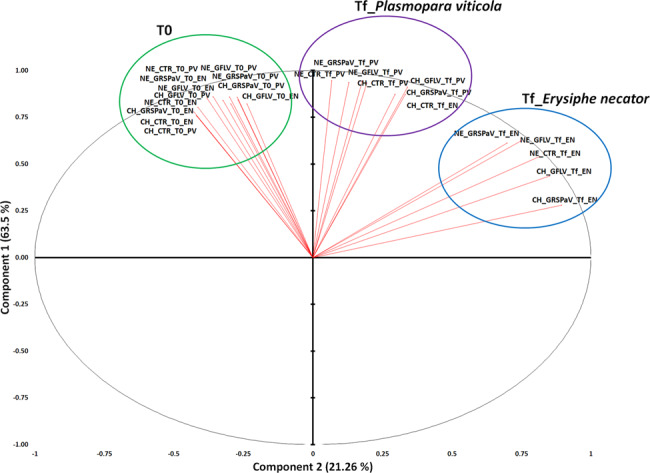
Principal component analysis (PCA) based on quantitative reverse transcription-polymerase chain reaction (RT-qPCR) values of the candidate genes reported in Table S1 analysed at T0 before inoculation with *P. viticola* (PV) or *E. necator* (EN) and at the end of experiments (Tf). NE_CTR, CH_CTR: virus-free plants; NE_GRSPaV, CH_GRSPaV: GRSPaV-infected plants; NE_GFLV, CH_GFLV: GFLV-infected plants. The first component (vertical) accounts for 63.5% of the variance and the second component (horizontal) accounts for 21.26%

The principal component analysis (PCA) of all RT-qPCR data showed division of the samples into three main groups (Fig. 4). Data at T0 were separated from those at Tf in plants inoculated with *P. viticola* and from the data at Tf in plants inoculated with *E. necator*. Among the factors that can determine the variability of these data, fungus/oomycete-mediated effects were those that determined the most relevant changes in the transcriptional regulation of selected genes. Only CH_CTR_Tf_EN behaved differently than the other samples infected by *E. necator*, since it grouped together with the Tf_PV data (Fig. 4).

## Discussion

### Set up of the experimental plan to study grapevine–virus interactions in controlled conditions

Over the past decade, studies on plant–virus interactions in grapevine have gained increasing importance^5–10,18^, suggesting that such species may be a suitable woody plant model for studies on this topic^11^. In these works, the molecular, metabolic, and physiological responses to virus infection varied considerably according to the viral strains, presence of multiple viral infections, grapevine genotype, and external environment, including climatic conditions. Starting from these considerations, we set up an experimental design aimed to control some of these variants to better understand whether and which kind of interactions existed between some viruses and *P. viticola* and *E. necator*, causal agents of DM and PM, respectively, in grapevine. The analyses were focused on GFLV and GRSPaV because both are widespread viruses, well known from the molecular point of view, and represent two extremes of the biological responses induced in grapevine. As a rule, GFLV is quite harmful to grapevine^3^, while GRSPaV is generally latent in *V. vinifera*. On the other hand, positive effects of interactions have been demonstrated in GRSPaV-infected plants subjected to drought^18^.

A critical point in our work was the strong influence of the grapevine genotype, so that healthy controls of the two cultivars, ‘Chardonnay’ and ‘Nebbiolo’, had to be produced by somatic embryogenesis^36,37^. These cultivars were chosen for their biological characteristics, e.g. different colour of the berries, different susceptibility to DM and PM, and for their importance and diffusion at international (‘Chardonnay’) and local levels (‘Nebbiolo’). In addition, as somatic embryogenesis may induce somaclonal variation^38^, the genetic uniformity of healthy and infected samples was guaranteed by using one single plant regenerated by a somatic embryo for each cultivar multiplied in vitro and infected with the selected viruses. Considering that the transmission of viruses to grapevine by mechanical inoculation is poor or not efficient at all, the grafting technique was adopted to transmit viruses from infected plants to virus-free ‘Chardonnay’ and ‘Nebbiolo’ cultivar.

### Changes in virus titre over time

The experimental plan highlighted the influence of the grapevine genotype and/or of the time factors on viral titre. The viral titre of GRSPaV was similar in ‘Nebbiolo’ and ‘Chardonnay’ and decreased at Tf, which is in line with the seasonal variation observed in leaves in the vineyard^6^. Notably, in’Nebbiolo’, GFLV titre in leaves was 5–6 times lower than in ‘Chardonnay’ during the whole sampling time of the experiment (Supplementary Fig. S3), and at Tf the titre decreased in both cultivars. Krebelj et al.^39^ reported that in vineyards, the highest GFLV titre was observed at the beginning of the vegetative period in May, and that the virus concentration changed in different grapevine cultivar. Our data agree with their study: the lower GFLV concentration at Tf may be considered a seasonal variation of virus titre, although decrease in viral titre may be caused by PM and DM infection. Viral accumulation is also affected by the grapevine genotype because both cultivars were infected with the same GFLV strain but differ in virus titre. Despite the differences in GFLV accumulation between ‘Nebbiolo’ and ‘Chardonnay’, no viral symptoms were observed throughout the experiments. This agreed with many years of observation of young potted grapevines in the greenhouse often not exhibiting viral symptoms (Gambino, personal observations).

### Powdery and downy mildews development

Under greenhouse conditions, grapevine-mediated interactions between the two viruses and both the DM and PM causal agents (artificially inoculated) were demonstrated. Although ‘Chardonnay’ and ‘Nebbiolo’ showed different susceptibility to these diseases, both cultivars infected with GFLV showed a greater tolerance to PM and DM. These results agree with previous observations, in which grapevines infected by GLRaV-3 in field conditions, were more tolerant to late infection of *P. viticola*^19^. Interestingly, the main differences were observed in the severity of PM and DM diseases. In both cultivars, GRSPaV did not induce significant variation in the development of DM and PM and providing DS and DI values that were generally intermediate between the virus-free controls and GFLV-infected plants. However, in the second trial, GRSPaV seemed to favour the development of DM with DS and DI slightly higher than virus-free controls, even of differences were not significant. Notably, plant responses induced by GRSPaV stimulate tolerance to water stress^18^, whereas they had no effect or worsened the presence to fungal/oomycete pathogens, as shown in this study.

### Target plant genes modulation in response to viruses and powdery and downy mildews

The complex tripartite interactions have not yet been sufficiently explored in terms of molecular and biochemical mechanisms activated to counteract the disease development. Plants evolved several strategies to control the immune system responses and limit the costs of resistance^40^. Although poorly explored in grapevine, the simultaneous presence of pathogens can lead the plants to develop a state of readiness or priming that enables earlier, faster and stronger defence responses to a subsequent attack as reported in other plant species^41^. To further explore these aspects, target genes were selected to investigate whether molecular defence priming can be activated in two different grape genotypes.

The first important factor inducing transcriptional modulation of the genes was the time (T0 and Tf) associated with inoculation of DM and PM causal agents. In some genes at Tf, the responses induced by *P. viticola* and *E. necator* were not always in line with the data reported for these pathogens^34,42,43^. This is because we chose to focus on the systemic responses induced by these biotrophic pathogens over a long period by sampling only asymptomatic leaves at Tf and we did not consider the early molecular responses in infected tissues, which were well characterized in DM^34,42^ and PM^43^. An example of this discrepancy was the regulation of *VvPR1* and *VvBgluc*, two genes codifying PR proteins, which are generally upregulated in the first days after the infection of *P. viticola*^42,44^ and *E. necator*^43,45^. These genes were downregulated at Tf in ‘Nebbiolo’ and substantially not modulated in ‘Chardonnay’, because the acute phase of both pathogen infection disappeared at Tf and the systemic activation of *VvPR1* and *VvBgluc* was likely switched off in asymptomatic tissues. This is supported by previous reports in ‘Sultana’ plants infected by *E. necator*, in which glucanase activity was concentrated in infected leaf areas, while activity in the uninfected areas of the same leaf was similar to those observed in healthy plants^45^. Stilbene synthases (*VvSTSs*) showed strong overexpression at Tf in all cases for both pathogens, and at Tf_EN these genes were expressed 3 to 10 times more than at Tf_PV. Stilbenes are the most important class of phytoalexins, which represent a powerful defence system against several pathogens, including *P. viticola* and *E. necator*^34,43,46^, and are key molecules of basal immunity in grapevine^47,48^. Defense responses involving *VvSTSs* persisted for a long period after inoculation with both pathogens, with higher levels after *E. necator* inoculation.

The grapevine genotype represents another relevant factor in the response to *P. viticol*a, *E. necator* and viruses and its effect was evident for some genes at T0. In detail, vacuolar invertase *VvGIN2* and *VvPR1*, and *VvBgluc*, the genes involved in stress responses, were constitutively expressed more in ‘Nebbiolo’ than in’Chardonnay’, supporting the previous findings on the specific interactions of ‘Nebbiolo’ with biotic stress^49^. In addition, these genes were strongly overexpressed in ‘Nebbiolo’ infected by GFLV and GRSPaV, which suggests that these viruses may activate some defence responses that primer plants more promptly to the infection by other pathogens.

The interactions between viruses and grapevine genotype significantly modified the susceptibility reaction of grapes to PM and DM. In carbohydrate metabolism, the expression of *VvGIN2* and *VvSUSY4* at T0 was downregulated in the presence of viruses, particularly in GFLV-infected plants. The induction of invertases and sucrose synthases can stimulate the change of leaves from source to sink organs^50^ and the accumulation of sugars can lead the plant tissues to host more easily the development of biotrophic pathogens, such as *P. viticola* and *E. necator*. Therefore, at T0, immediately before the fungal/oomycete inoculation, virus-free plants (NE_CTR and CH_CTR) with high expression of *VvGIN2* and *VvSUSY4* seem to be more suitable for the development of these pathogens compared to virus-infected plants, as confirmed by the DS data. At Tf, after inoculation with *P. viticola* and *E. necator*, systemic activation of these genes in asymptomatic leaves was not observed, as conversely reported in symptomatic tissues^51^, suggesting a specific role(s) in symptomatic tissues to counteract pathogen development. The sucrose transporters *VvSTP13* and *VvSUC27* were upregulated at Tf after *E. necator* inoculation and, in ‘Chardonnay’, virus infection significantly increased this upregulation, particularly *VvSUC27* in CH_GFLV_Tf_EN. In *Arabidopsis*, the high constitutive level of *STP13* led to an enhanced basal resistance to *Botrytis cinerea*^52^, while in grapevine, high transcription levels of *VvSTP13* were induced by the rootstocks in scions that were more tolerant to DM^53^. In addition, the transcriptional increase of *VvSUC27* associated with the downregulation of *VvGIN2* is one of the responses observed in the resistant species *V. amurensis* after *P. viticola* inoculation^54^. Therefore, the similar regulation observed in GFLV-infected ‘Chardonnay’ could partially explain the increased tolerance to DM and PM.

All these findings, at least in our experimental controlled conditions, suggest a virus primed defence state that increases resilience against fungal/oomycete pathogens.

## Conclusions

In this study, infection by GFLV reduced the disease severity caused by *E. necator* and *P. viticola* in grapevine. GFLV infection caused the overexpression of stilbene synthase genes and pathogenesis related proteins (*VvPR1*, *VvBgluc*) and influenced carbohydrate metabolism thus modifying the expression dynamics of sugar transporters (*VvSTP13*, *VvSUC27*), vacuolar invertase, and sucrose synthase (*VvGIN2* and *VvSUSY4*) genes. These transcriptional changes can trigger the synthesis of defence compounds, improving the innate plant immune response in grapevine and making the plant prompter to respond to biotic stress. However, this does not appear to be a generic reaction to all grapevine viruses, as in GRSPaV-infected plants, the transcriptional modulation of candidate genes was not sufficient to promote protection against *E. necator* and *P. viticola*. It is likely that this increased tolerance to fungus/oomycete is an indirect effect of plant responses to viruses, such as GFLV and GLRaV-3^19^. Additionally, ‘Nebbiolo’ showed a higher constitutive expression of some genes in response to pathogen compared to ‘Chardonnay’, confirming previous observations by Gambino et al.^49^. However, the hypothetical application of GFLV-infected plants within integrated pest management strategies is not feasible, because the GFLV is a harmful virus that must be absent in the grapevine propagation material according to the certification protocols. It will be interesting to broaden our understanding of these interactions which can also be investigated under field conditions for several years, and in the presence of multiple virus and fungal/oomycete infections.

## Materials and methods

### Plant material

Virus-free plantlets of *V. vinifera* cv. Nebbiolo and Chardonnay were regenerated from somatic embryos obtained from in vitro culture of immature anthers collected in spring (May 2016), according to the previously described protocol^36^. The regenerated plantlets were micropropagated by sub-culturing apical cuttings thus giving rise to individual lines^55^. Virus and viroid infections were assessed by RT-PCR assays as described below. A single virus-free line originated from a single somatic embryo was selected, multiplied, and used for subsequent analyses.

GFLV or GRSPaV were transmitted to virus-free ‘Nebbiolo’ and ‘Chardonnay’ lines by in vitro grafting. Woody cuttings from plants infected only by GFLV or GRSPaV were collected during winter and forced to sprout in water at room temperature. Herbaceous green branches (about 5 cm) emerging from the buds were collected, surface-sterilized for 15 min with sodium hypochlorite (1.5% available chlorine) and rinsed several times with sterile distilled water. Ten apical cuttings (3–4 cm long) for each virus-free line were grafted on virus-infected herbaceous green branches and maintained under in vitro conditions. After 1 month, the virus infection was assessed by RT-PCR and a single apical cutting of ‘Nebbiolo’ and ‘Chardonnay’ infected by GFLV or GRSPaV were micropropagated by repetitively sub-culturing apical cuttings.

For each thesis, ‘Nebbiolo’ and ‘Chardonnay’ virus-free (NE_CTR, CH_CTR), infected by GFLV (NE_GFLV, CH_GFLV), and infected by GRSPaV (NE_GRSPaV, CH_GRSPaV), 16 plantlets were acclimated out of in vitro conditions in 1 litre pots (10 × 10 × 12 cm) using a peat substrate (TS4, Turco Silvestro, Italy) and placed in the greenhouse (Supplementary Fig. S1).

### Virus detection

The major viruses and viroids commonly present in wine grape cultivar grown in north western Italy are GFLV, GVB, grapevine fleck virus (GFkV), grapevine leafroll associated virus-1, -2, -3 (GLRaV-1, -2, -3), grapevine–virus A (GVA), arabis mosaic virus (ArMV) and GRSPaV. These viruses were detected by multiplex RT-PCR reported in Gambino^56^, as well as grapevine pinot gris virus (GPGV) following the protocol of Glasa et al.^57^. Multiplex RT-PCR assays reported by Hajizadeh et al.^58^ were adopted for the detection of five viroids: hop stunt viroid (HSVd), grapevine yellow speckle viroid 1 and 2 (GYSVd-1, -2), australian grapevine viroid (AGVd), and citrus exocortis viroid (CEVd). One accession of ‘Nebbiolo’, originally infected by GFLV and GRSPaV, was previously subjected to sanitation. The in vitro thermotherapy and meristem tip culture sanitation techniques were used to eliminate GFLV from some explants and GRSPaV from other ones, thus obtaining some lines still infected by only one of these two viruses, as confirmed by RT-PCR. The two plants were used for the grafting experiments described above.

To characterize the isolates of the two viruses, RT-PCR amplification products were purified and sequenced by Sanger sequencing, as reported previously^49^. PCR was carried out using Phusion Hot Start II High-Fidelity DNA Polymerase (Thermo Fisher Scientific, Waltham, MA USA) according to manufacturer’s instructions. For GFLV, primers GFLVpoly5238Fw and GFLVpoly6048Rev reported by Pacifico et al.^33^ targeting 829 bp of the putative RNA-dependent RNA polymerase (RdRp) (1E^Pol^) gene localized in RNA1 of the virus were used (Supplementary Table S1). For GRSPaV, primers RSP35 and RSP36^31^ targeting 476 bp of the genomic region encoding the RdRp domain were used (Supplementary Table S1). A total of 107 sequences of GFLV RNA1 and 123 sequences of GRSPaV were deposited in GenBank (National Center for Biotechnology Information, NCBI) and aligned with the respective ‘Nebbiolo’ isolates using Multiple Sequence Alignment (MUSCLE, https://www.ebi.ac.uk/Tools/msa/muscle/). Phylogenetic analysis based on the neighbour-joining (NJ) method (with bootstrap values of 1000 replicates) was carried out using MEGAX software version 10.1.7^59^.

### Experimental layout and inoculation of *P. viticola* and *E. necator*

Two independent trials were carried out in the greenhouse at the University of Turin, Agroinnova Competence Center, located in Grugliasco, in the Northwest of Italy (GPS: 45° 03’ 57.8” N, 7° 35’ 29.5” E) in summer 2018 and 2019. For each trial and pathogen (*P. viticola* and *E. necator*), four replicates (two plant/replicate) with a randomized block design were used for each infected or virus-free plant for both genotypes. Three months after growing in greenhouse conditions, the plants were artificially inoculated with *P. viticola* and *E. necator* collected in Piedmont (Northern Italy). The infected leaves with *P. viticola* from ‘Chardonnay’ plants and of *E. necator* from ‘Moscato’ plants were shaken in 100 ml of sterile deionized water, and each suspension obtained was adjusted, with the aid of a haemocytometer, to 5 × 10^3^ sporangia/ml and 1 × 10^4^ conidia/ml, respectively. The artificial inoculation of the pathogens was carried out for each trial throughout nebulisation with a laboratory spray bottle (10 ml of capacity) of one ml of suspensions/plant. All plants artificially inoculated with *P. viticola* or *E. necator* were maintained in two different greenhouse compartments in order to avoid cross contamination, at temperatures ranging from 20 and 25 °C and relative humidity maintained close to 85–90% for *P. viticola* and 25–27 °C for *E. necator* without overhead irrigation. The dates of the different operations carried out are reported under Supplementary Table S3.

About 10–25 leaves/plant were visually estimated by rating the percentage of affected leaves (disease incidence, DI) for both pathogens. The leaf area affected by the pathogen (disease severity, DS) was estimated using the severity scale 1–7 corresponding to 1= no symptoms; 2 < 5%; 3 = 5–10% 4 = 10–25%; 5 = 25–50%; 6 = 50–75% and 7 > 75% as reported by the EPPO / OEPP protocols for *P. viticola* (PP 1.31) or *E. necator* (PP1.4) by visually check 10 to 25 leaves / plant for each plant. DS was calculated using the formula: ∑(number of leaves × rating scale 0–7) / (total number of recorded leaves).

### Quantitative RT-PCR analysis

Fifteen genes representative of the most important molecular pathways involved in the response to biotic agents in grapevine were analysed (Supplementary Table S1). In the second fungal/oomycete inoculation trial in 2019, for each cultivar (‘Nebbiolo’ and ‘Chardonnay’), viral condition (CTR not infected, GFLV-infected plants, and GRSPaV-infected plants), and fungal/oomycete inoculated (*P. viticola* and *E. necator*), leaves were collected before the fungal inoculation (T0) and on the day of the final assessment of DS and DI of fungal/oomycete diseases (Tf) from three biological replicates for a total of 72 samples (2 genotypes × 3 viral conditions × 2 fungal/oomycete pathogen × 2 collections × 3 biological replicates) (Supplementary Fig. S1). For each biological replicate, fully developed leaves (without fungal symptoms for the Tf) in the middle of shoots were collected from two or three plants and stored at −80 °C. Total RNA was extracted using the rapid CTAB method^60^ and RNA quantity and quality were checked using a NanoDrop 1000 spectrophotometer (Thermo Fisher Scientific, Waltham, MA, USA). DNase treatment, first-strand cDNA synthesis, and real-time PCR were carried out as reported by Chitarra et al.^53^. The results were calculated as expression ratios (relative quantity, RQ) relative to NE_CTR at T0.

Relative quantification of GFLV and GRSPaV were carried out on leaves collected at T0 and Tf using primers designed with viral RdRp (Supplementary Table S1) and following the same procedure reported above.

### Statistical analyses

DI and DS were analyzed using SPSS software 26. The DI and DS data were subjected to Levene’s test to determine the homogeneity of the variance and to Shapiro–Wilk test to check the normality. When necessary DI and DS data, were arc-sin-transformed to stabilize the variances and normalize their distribution. The General linear model analysis was conducted to test for the effect of each factor (genotype and virus infection), and their interactions on DI and DS. When the effects of the tested factors were significant (*p* ≤ 0.05) and interactions were observed among the considered factors, the means were separated by Tukey’s honestly significant difference test (HSD) at *p* ≤ 0.05.

For RT-qPCR data, when ANOVA indicated that either genotype (NE, CH), virus (CTR, GRSPaV, GFLV), and time (T0, Tf) factors or their interaction was significant, mean separation was performed using the Tukey’s HSD test at a probability level of *p* ≤ 0.05. The standard deviation (SD) of all means were calculated.

## Supplementary information

Supplementary material

Supplementary Table S1

Supplementary Table S2

## Data Availability

The sequences of the virus isolates used in this study are available at NCBI accession numbers MN889892 (GRSPaV_NE) and MN889891 (GFLV_NE).
