# Supplementary material for: Biological and molecular interplay between two viruses and powdery and downy mildews in two grapevine cultivars
Source: Hortic Res. 2020 Nov 1;7:188. doi: 10.1038/s41438-020-00413-x (PMC7603506; doi:10.1038/s41438-020-00413-x)
Supplement: Supplementary file 1 — Supplementary material [file 41438_2020_413_MOESM1_ESM.pdf]

Biological and molecular interplay between two viruses and powdery and downy mildews in two grapevine cultivars

Giovanna Gilardi, Walter Chitarra, Amedeo Moine, Monica Mezzalama, Paolo Boccacci, Massimo Pugliese, Maria Lodovica Gullino, Giorgio Gambino*

SUPPORTING INFORMATION

Table S3. Date of the fungus/oomycete inoculation trials.

Operation	*Plasmopara viticola*		*Erysiphe necator*	
	**Trial 1**	**Trial 2**	**Trial 1**	**Trial 2**
**T0 sampling**		10/06/2019		10/06/2019
**Artificial inoculation**	19/06/2018	10/06/2019	20/08/2018	10/06/2019
**Final assessment**	19/07/2018	05/07/2019	05/10/2018	05/07/2019
**Tf sampling**		05/07/2019		05/07/2019
**Number of plants per thesis**	8	8	8	8
**Blocks**	4	4	4	4

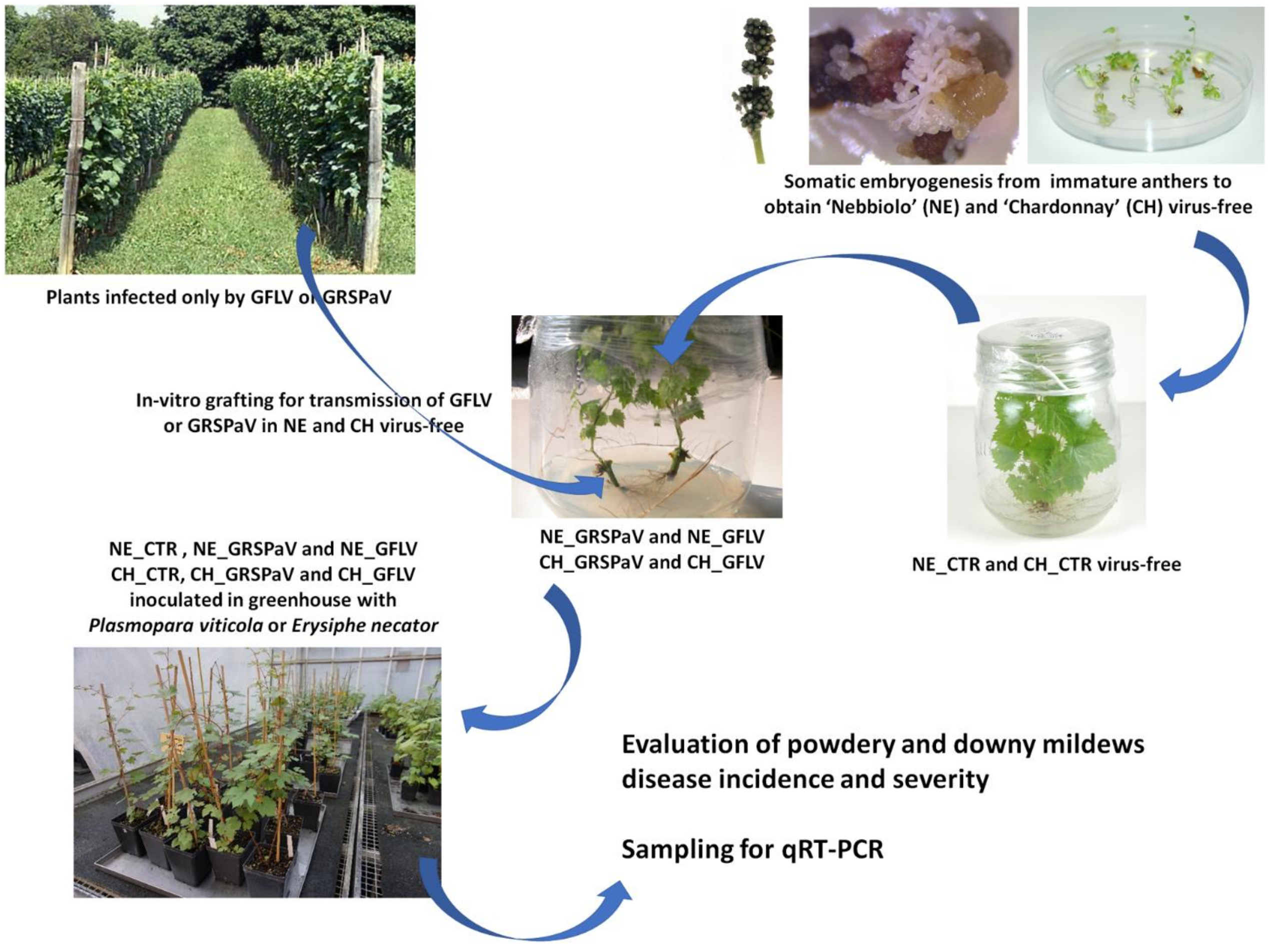

Figure S1. Graphic description of the experimental plan adopted in this work. Grapevines infected with GRSPaV or GFLV identified in the field, were grafted *in vitro* with virus-free plants of 'Chardonnay' (CH_CTR) and 'Nebbiolo' (NE_CTR) regenerated by somatic embryogenesis. Virus-free (NE_CTR and CH_CTR) and infected plants (NE_GRSPaV, NE_GFLV, CH_GRSPaV, CH_GFLV) were acclimated to the greenhouse for artificial inoculation with *P. vitcola* or *E. necator*. Before fungal/oomycete inoculation (T0) and at the end of experiment, when assessing the incidence of the diseases and the severity of the attacks by fungal pathogens (Tf), the leaves were sampled for qRT-PCR, which was performed on some candidate genes.

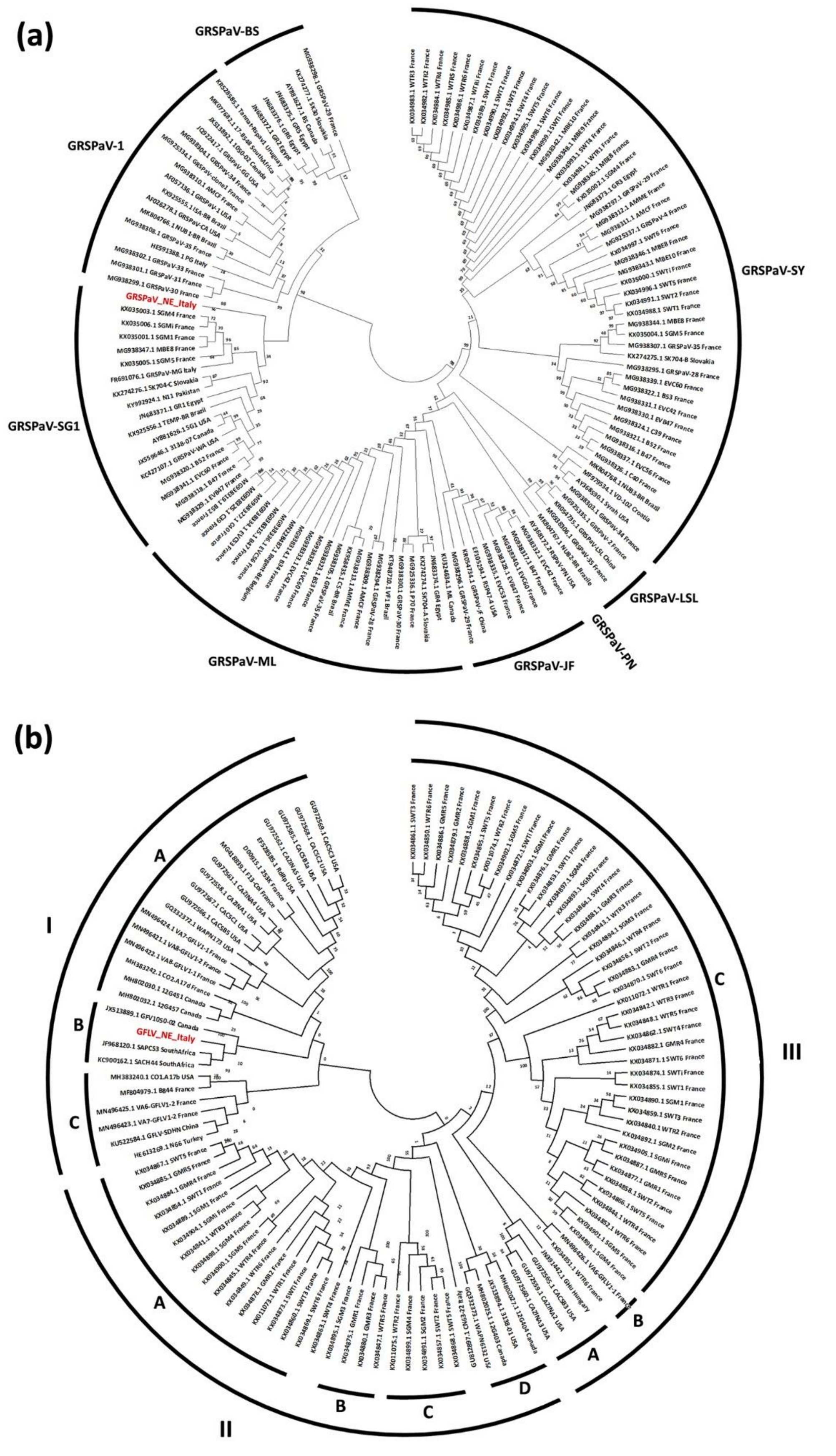

Figure S2. Phylogenetic trees of GRSPaV and GFLV sequence variants. **a**) The Neighbour–Joining tree of the GRSPaV region corresponding to putative viral RNA-dependent RNA polymerase (RdRp).

The NCBI accession numbers, names, and country of origin of the GRSPaV isolates are reported in the phylogenetic tree. The GRSPaV isolate infecting ‘Nebbiolo’ is marked in red (NCBI accession number MN889892). Clusters are designated by the name of the reference isolate (Meng and Rowhani, 2017). **b**) The Neighbour–Joining tree of the GFLV region corresponding to putative viral RNA-dependent RNA polymerase (RdRp) in RNA1. The NCBI accession numbers, names, and country of origin of the GFLV isolates are reported in the phylogenetic tree. The GFLV isolate infecting Nebbiolo is marked in red (NCBI accession number MN889891). In both phylogenetic trees, the robustness of each node was tested using 1000 bootstrap replicates.

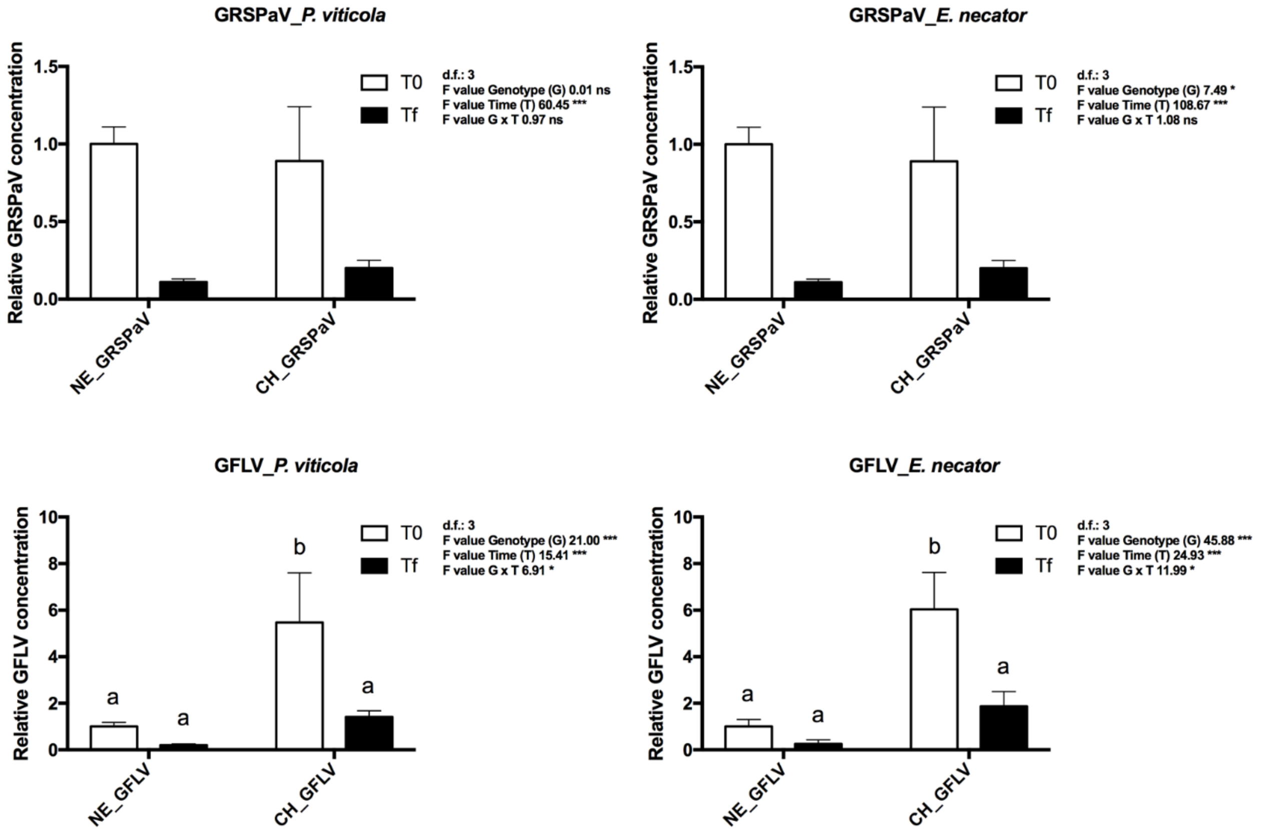

Figure S3. Quantification of GRSPaV and GFLV RNA in leaf of ‘Nebbiolo’ (NE) and ‘Chardonnay’ (CH) as determined by quantitative reverse transcription-polymerase chain reaction (qRT-PCR). Samples were collected before the inoculation of *P. viticola* or *E. necator* (T0) and at the end of experiments (Tf). qRT-PCR signals were normalized to *VvAct* and *VvUBI* transcripts. NE_CTR, CH_CTR: virus-free plants; NE_GRSPaV, CH_GRSPaV: GRSPaV-infected plants; NE_GFLV, CH_GFVL: GFLV-infected plants. Data are presented as the mean ± standard deviation (SD) (n = 3). Lowercase letters denote significant differences attested by Tukey’s honestly significant difference (HSD) test ($P < 0.05$).

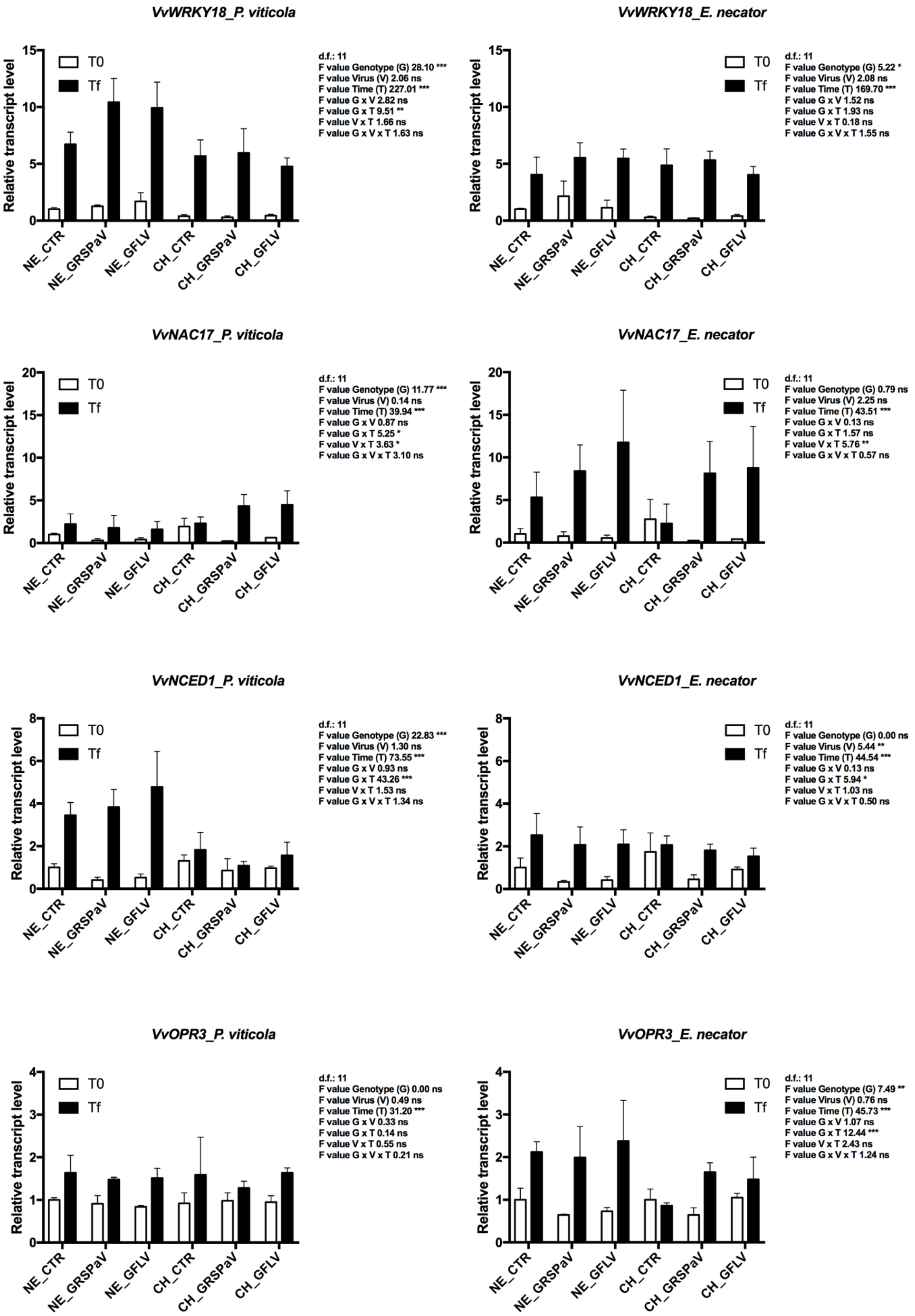

Figure S4. Relative expression levels of *VvWRKY18* (VIT_04s0008g05760), *VvNAC102* (VIT_19s0014g03290), *VvNCED1* (VIT_19s0093g00550), and *VvOPR3* (VIT_11s0016g01230)

measured by quantitative reverse transcription-polymerase chain reaction (RT-PCR). Samples were collected before inoculation with *P. viticola* or *E. necator* (T0) and at the end of experiments (Tf). qRT-PCR signals were normalized to *VvAct* and *VvUBI* transcripts. NE_CTR, CH_CTR: virus-free plants; NE_GRSPaV, CH_GRSPaV: GRSPaV-infected plants; NE_GFLV, CH_GFVL: GFLV-infected plants. Data are presented as the mean ± standard deviation (SD) (n = 3). Lowercase letters denote significant differences attested by Tukey's honestly significant difference (HSD) test ($p \leq 0.05$).
